# The Relative Impacts of Climate and Land-Use Change on Conterminous United States Bird Species from 2001 to 2075

**DOI:** 10.1371/journal.pone.0112251

**Published:** 2014-11-05

**Authors:** Terry L. Sohl

**Affiliations:** Earth Resources Observation and Science (EROS) Center, U.S. Geological Survey, Sioux Falls, South Dakota, United States of America; U.S. Geological Survey, United States of America

## Abstract

Species distribution models often use climate data to assess contemporary and/or future ranges for animal or plant species. Land use and land cover (LULC) data are important predictor variables for determining species range, yet are rarely used when modeling future distributions. In this study, maximum entropy modeling was used to construct species distribution maps for 50 North American bird species to determine relative contributions of climate and LULC for contemporary (2001) and future (2075) time periods. Species presence data were used as a dependent variable, while climate, LULC, and topographic data were used as predictor variables. Results varied by species, but in general, measures of model fit for 2001 indicated significantly poorer fit when either climate or LULC data were excluded from model simulations. Climate covariates provided a higher contribution to 2001 model results than did LULC variables, although both categories of variables strongly contributed. The area deemed to be “suitable” for 2001 species presence was strongly affected by the choice of model covariates, with significantly larger ranges predicted when LULC was excluded as a covariate. Changes in species ranges for 2075 indicate much larger overall range changes due to projected climate change than due to projected LULC change. However, the choice of study area impacted results for both current and projected model applications, with truncation of actual species ranges resulting in lower model fit scores and increased difficulty in interpreting covariate impacts on species range. Results indicate species-specific response to climate and LULC variables; however, both climate and LULC variables clearly are important for modeling both contemporary and potential future species ranges.

## Introduction

Species distribution models (SDMs) are based on the assumption that presence at a given location is based on suitable environmental conditions to support the species' ability to find shelter, feed, and/or reproduce [1], [2]. Such models have been widely used to model current species distributions, either to establish extant distributions or to understand the specific environmental variables that drive species distributions [3], [4], [5]. A central premise of many SDMs is that climate is a primary driving force of the distribution of species [6]. Projected climate data are frequently used with SDMs to explore potential future impacts of climate change on species distributions [7], [8], [9], based on the assumption that the basic physiological tolerances of species to environmental conditions are constant through time [10]. Jimenez-Valverde et al. [11] modeled typical climate conditions for 94 bird species in North America and noted the dominant signal of climate in shaping North American bird distributions. Thuiller et al. [12] modeled distributions of plants, birds, mammals, and reptiles in Europe and found that models using climate alone performed nearly as well as models that included both climate and landscape variables. Bucklin et al. [13] found that climate variables were strong predictors for contemporary species distribution modeling and that additional predictors (including land cover) were not essential.

Climate is obviously a primary driver of many SDMs. While land use and land cover (LULC) change is often used for modeling contemporary species distributions, it is not often used when examining future time frames [7]. Despite the results from the studies listed above, other studies have found that including LULC in bioclimatic models of species distribution can improve the explanatory power of SDMs [7], [12]. Lee and Jetz [14] found that LULC projections were vital for future modeling, noting that loss of habitat is a high predictor of extinction for bird species. Barbet-Massin et al. [7] found that SDMs perform best if both climate and LULC are included. Sinclair et al. [15] were critical of SDMs for rarely including anthropogenic impacts on biological systems, suggesting that changing landscape patterns are likely to have at least as great an impact on species distributions as climate change. Many other studies have found that projected land use is a vital component of SDMs, with loss of predictive power when LULC is not included in the assessment [1], [8], [9], [16].

While many state the need to use LULC data when projecting future change in species distributions, such data are often not available [17]. Riordan et al. [16] noted the disconnect between relatively high-resolution climate projections used in global assessment models and the generally coarse treatment of LULC, leading to high-resolution, projected LULC rarely being used (or available) for SDMs. Those studies that have used both projected climate and LULC data to model future species distributions have often relied on very coarse spatial-scale LULC data [1], [7]. LULC is much more heterogeneous at local scales than climate. Those studies that do use LULC but only at very coarse scales miss the inherent spatial variability in LULC that is typically not found with climate data.

The goals of this paper are to examine the relative effects of climate and LULC change on bird species distributions in the conterminous United States, using maximum entropy modeling and projected climate and LULC data from 2001 to 2075. Specific research questions include:

- What are the relative influences of LULC and climate in modeling contemporary (2001) breeding bird distributions for the conterminous United States?
- What are the relative impacts of projected climate and projected LULC change on United States breeding bird distributions in the future (2075)?
- What are the specific impacts of climate and LULC on one focus species (Hooded Warbler (*Wilsonia citrina*) used to demonstrate individual species results)?
- What are the implications for the use of climate and LULC data in SDMs?

## Materials and Methods

A maximum entropy model (Maxent) was used in conjunction with species presence data, current and projected climate and LULC data, and topographic data to model distributions for 50 diverse bird species in the conterminous United States. Twelve distinct modeling simulations were conducted for each species to disentangle the effects of climate and LULC on species distributions for both the “present” (2001 for this assessment) and for multiple scenarios in the future (2075). Although many supporting data were available through the year 2100, 2075 was selected as the assessed “future” year to accommodate the use of 30-year averaged climate data (as described below). The following provides a summary of data sources, the model structure, model parameterization, and the assessment framework.

### Materials

The modeling approach described below required spatially explicit data on both bird species “presence”, as well as environmental variables (covariates) that could be used to model species distributions. Note the goal was to assess long-term trends in changes to species distributions. Longer-term aggregate or average data values were thus used to temper the effects of seasonal or yearly variation, both for the species presence data (where distributions of single-season presence records could be impacted by unusual seasonal conditions such as drought or large disturbance events) and for the covariates (where single-season climate data, for example, may be unrepresentative of longer-term climate trends).

#### Species Presence Data – eBird

The source of bird species presence data was eBird, a “citizen-science” database [18], [19]. eBird allows public entry of bird sightings, with recorded information on the time and date of the sighting, location, observation protocol, quantity of each species, and observer information. As a citizen science database, there are potential issues (discussed below) related to the lack of a formal sampling protocol [20], but eBird offers several potential advantages for species distribution modeling including 1) large number of sample points (millions for some species), 2) global data (although observations are currently heavily biased towards North America and Europe), and 3) observations for all seasons. eBird data have been successfully used for a number of SDM assessments [20], [21], [22], [23]. Hochachka and Fink [20] found strong linkages between individual species and land cover using eBird data, and that the data were valuable for examining distribution patterns at multiple scales.

Fifty bird species with breeding ranges partially or completely within the conterminous United States were selected for the assessment (**Table 1**). Species were selected to ensure variability in size of breeding range, geographic region, and preferred breeding habitat. The goal was to ensure that a variety of “real-world” model applications were represented. To minimize potential effects of annual variation in species presence, data from eBird entries from 1992 to 2012 were used to establish “current” breeding records. With current and projected land cover data available for every year from 1992 to 2100 (see below), a nominal date of 2001 (middle of the 1992 to 2010 period) was used to represent contemporary species distributions and tie the 1992 to 2010 species occurrences to one specific date of land-cover conditions. Data were also filtered by season to ensure records corresponded to breeding populations; for all species a consistent June 1 to July 15 observation period was used to represent likely “breeding” presence, a reasonable assumption for the species that were assessed. Some migratory species initially included in the assessment were removed from consideration based on dispersed patterns of eBird sightings for the June 1 to July 15 period, indicating post-breeding movement had already occurred by July 15 (e.g., Long-billed Curlew (*Numenius americanus*)). One species included, the American Goldfinch (*Spinus tristis*) generally begins breeding after this period, but is considered non-migratory and still is within breeding range. For the 50 species assessed, eBird sightings for the June 1 to July 15 period corresponded well to published breeding range maps from NatureServe [23].

**Table 1:** Species modeled and number of eBird sample points for each.

	Species	Scientific Name	Original	Final
**1**	**American Goldfinch**	*Spinus tristis*	236,217	2,663
**2**	**Anna’s Hummingbird**	*Calypte anna*	32,047	427
**3**	**Baird’s Sparrow**	*Ammodramus bairdii*	513	48
**4**	**Band-tailed Pigeon**	*Patagioenas fasciata*	17,415	407
**5**	**Black-capped Chickadee**	*Poecile atricapillus*	131,634	1,877
**6**	**Blue-winged Teal**	*Anas discors*	15,288	1,243
**7**	**Bobolink**	*Dolichonyx oryzivorus*	28,658	1,105
**8**	**Brown-headed Cowbird**	*Molothrus ater*	178,324	3,996
**9**	**Brown Thrasher**	*Toxostoma rufum*	61,661	2,254
**10**	**Cactus Wren**	*Campylorhynchus brunneicapillus*	4,714	215
**11**	**Carolina Wren**	*Thryothorus ludovicianus*	107,244	1,893
**12**	**Chestnut-collared Longspur**	*Calcarius ornatus*	1,426	105
**13**	**Dickcissel**	*Spiza americana*	29,479	1,411
**14**	**Downy Woodpecker**	*Picoides pubescens*	150,261	2,925
**15**	**Eastern Kingbird**	*Tyrannus tyrannus*	111,057	2,956
**16**	**Ferruginous Hawk**	*Buteo regalis*	1,587	238
**17**	**Gambel’s Quail**	*Callipepla gambelii*	6,307	198
**18**	**Grasshopper Sparrow**	*Ammodramus savannarum*	23,254	1,323
**19**	**Gray Partridge**	*Perdix perdix*	616	129
**20**	**Gray Vireo**	*Vireo vicinior*	265	43
**21**	**Great Blue Heron**	*Ardea herodias*	141,552	3,449
**22**	**Great Horned Owl**	*Bubo virginianus*	11,130	1,487
**23**	**Green-winged Teal**	*Anas carolinensis*	6,726	530
**24**	**Hooded Warbler**	*Wilsonia citrina*	15,482	773
**25**	**Lark Bunting**	*Calamospiza melanocorys*	3,268	355
**26**	**Lark Sparrow**	*Chondestes grammacus*	20,978	1,467
**27**	**Northern Harrier**	*Circus cyaneus*	14,795	1,231
**28**	**Northern Pintail**	*Anas acuta*	4,269	466
**29**	**Orchard Oriole**	*Icterus spurius*	41,136	1,876
**30**	**Painting Bunting**	*Passerina ciris*	15,294	569
**31**	**Pied-billed Grebe**	*Podilymbus podiceps*	23,272	1,287
**32**	**Pileated Woodpecker**	*Dryocopus pileatus*	48,118	1,982
**33**	**Pygmy Nuthatch**	*Sitta pygmaea*	11,848	322
**34**	**Red-eyed Vireo**	*Vireo olivaceus*	138,887	2,389
**35**	**Red-headed Woodpecker**	*Melanerpes erythrocephalus*	22,809	1,593
**36**	**Red-tailed Hawk**	*Buteo jamaicensis*	101,388	3,715
**37**	**Ruby-throated Hummingbird**	*Archilochus colubris*	81,241	2,090
**38**	**Savannah Sparrow**	*Passerculus sandwichensis*	41,214	1,435
**39**	**Scissor-tailed Flycatcher**	*Tyrannus forficatus*	16,571	718
**40**	**Sedge Wren**	*Cistothorus platensis*	7,827	478
**41**	**Sharp-tailed Grouse**	*Tympanuchus phasianellus*	801	121
**42**	**Short-eared Owl**	*Asio flammeus*	760	139
**43**	**Sora**	*Porzana carolina*	6,687	649
**44**	**Tufted Titmouse**	*Baeolophus bicolor*	129,472	2,058
**45**	**Vesper Sparrow**	*Pooecetes gramineus*	16,387	1,164
**46**	**Western Kingbird**	*Tyrannus verticalis*	45,319	2,028
**47**	**Western Meadowlark**	*Sturnella neglecta*	36,755	1,825
**48**	**Western Tanager**	*Piranga ludoviciana*	33,108	1,127
**49**	**White-headed Woodpecker**	*Picoides albolarvatus*	4,389	137
**50**	**Yellow-headed Blackbird**	*Xanthocephalus xanthocephalus*	16,794	1,031

“Original” represents conterminous United States observations from 1992 to 2012, from June 1 to July 15. “Final” represents points that have had 1) spatial filtering applied to reduce points in heavily sampled areas, and 2) removal of points with long travel distances (traveling count) or large search areas (search area count).

eBird allows users to enter one of several potential observation protocols, including “stationary count”, “traveling count”, or “exhaustive area count”. However, regardless of observation protocol, users only enter one geographic coordinate. A single coordinate for a “traveling count” where the travel distance was substantial could result in a data point that was many kilometers from the actual observation. For exhaustive area counts with a large search area, a single coordinate may similarly be some distance from the actual observation. To eliminate potential issues with unrepresentative locations of eBird sightings, all “traveling count” sightings with a travel distance of more than 2 km were eliminated (similar to Fink et al. [23]), as were all “exhaustive area count” sightings with a search area of more than 100 hectares.

Additional potential issues with eBird data include spatial bias in presence samples [25]. eBird observations, like other citizen science data, tend to be clustered around highly populated and/or easily accessible areas [15], [18], [19]. Sampling bias has a much stronger effect on presence-only models (used here) than on presence-absence models, as model results end up representing both presence, as well as the density of the sampling effort [26]. Spatial filtering is an effective means to reduce bias in sample data prior to use in species distribution modeling [27], [28]. For this assessment, the seasonal 1992 to 2012 observations were spatially filtered to eliminate sample points within 20 km of any other sample point. The threshold of 20 km was chosen because it more aggressively reduced sampling density in the very dense eBird database than past studies [27], [28], while still maintaining adequate numbers of points for modeling. The elimination of sample points based on observation protocol or sampling density greatly reduced the number of sample points used in the assessment, often by a factor of 20 or more (**Table 1**). However, the filtering successfully eliminated the high concentration of points in heavily populated areas while maintaining a relatively large number of observations for most species (minimum of 43 points, maximum of 3,996, mean of 1,313). Only two species had fewer than 100 sample points (Gray Vireo (*Vireo vicinior*) and Baird’s Sparrow (*Ammodramus bairdii*)), at 48 and 43 points, respectively. The number of sample points were considered adequate, as Wisz et al. [29] and Hernandez et al. [30] examined the effect of sample size on species distribution models and found that Maxent outperformed other modeling techniques when sample sizes were small, with “reasonable” models possible with sample sizes as small as 10.

#### Land-Use and Land-Cover Data

A newly available suite of LULC projections for the conterminous United States was used [31], [32]. The LULC projections were produced for the conterminous United States, with annual LULC maps from 1992 to 2100 for four Intergovernmental Panel on Climate Change (IPCC) Special Report on Emissions Scenarios (SRES) [33]. The spatial resolution of the data was 250 m, with 16 LULC classes. The four modeled SRES were the A1B, A2, B1, and B2 scenarios; however, complimentary climate data were not available for the B2 scenario, so only A1B, A2, and B1 were used in this assessment (see **table 2** for characteristics of the three IPCC SRES scenarios used in this assessment). To simplify the modeling and interpretation of model results, the original sixteen LULC classes were aggregated to eight basic LULC classes (**table 3**). Aggregated 2001 LULC data served as one of the covariates when constructing the initial models. Projected 2075 LULC data provided information on LULC change for the 2075 model simulations.

**Table 2:** Relative socioeconomic characteristics of the three IPCC SRES scenarios used in this assessment.

	A1B	A2	B1
**Primary focus**	Economic growth	Economic growth	Environmental sustainability
**Globalization or** **Regionalization**	Global Convergence	Regional Development	Global Convergence
**Global Population**	Increase to 8.7 billion by2050, then slow decline	Continuous increase to15.1 billion by 2100	Increase to 8.7 billion by 2050, then slow decline
**Gross Domestic** **Product Growth**	Very High	Medium	High
**Energy Use**	Very High	High	Low
**Energy Strategy**	Balanced, fossil fueland alternative fuels	Regionally variable,based on local resources	Push to alternative and post-fossil fuel energy
**Pace of technology** **change**	Rapid	Slow	Medium
**Technology** **diffusion**	Rapid	Slow, regional variability	Rapid
**Economic equity**	Homogenization, higherincomes	Fragmented, uneven,continued income gaps	Homogenization, but lower incomes than A1B
**Environmental** **Protection**	Focus on “management”of resources rather than“conservation”	Uneven environmentalmanagement, protectionhigher in affluent areas	Broad support for environmental conservation, efficiency gains for resource use

See Nakicenovic et al. (2000) for additional information on SRES characteristics and Sohl et al. (2014) for how these characteristics were interpreted to create the LULC projections used in this assessment.

**Table 3:** Covariates used as predictor variables within Maxent.

Variable Category	Variable Name	Description
Land Cover	Cropland Count	5×5 neighborhood count of “cropland” pixels
Land Cover	Forest Count	5×5 neighborhood count of “forest” pixels (all forest)
Land Cover	Grass Count	5×5 neighborhood count of “grassland” pixels
Land Cover	Hay Count	5×5 neighborhood count of “hay/pasture” pixels
Land Cover	Shrub Count	5×5 neighborhood count of “shrubland” pixels
Land Cover	Urban Count	5×5 neighborhood count of “urban” pixels
Land Cover	Water Count	5×5 neighborhood count of “water” pixels
Land Cover	Wetland Count	5×5 neighborhood count of “wetland” pixels (all wetland)
Land Cover	LULC Diversity	5×5 neighborhood count of the number of different LULC classes
Climate	Average Temp	Average annual temperature
Climate	Average Precip	Average annual (total) precipitation
Topography	Elevation	Elevation data from National Elevation Database
Topography	Slope	Slope data derived from National Elevation Database
Topography	Compound Topographic Index	Compound Topographic Index data derived from National Elevation Database

All data were mapped to a common geographic extent at 250-m resolution.

Error in LULC data obtained from remote sensing sources is a concern for SDMs [4]. The LULC projections described above used the 1992 National Land Cover Database (NLCD) [34] as the mapping starting point. The projections were thus subject to not only the inherent uncertainty in projecting future LULC conditions, but also carried the legacy of any mapping error in the original 1992 NLCD. Given the lack of a rigid sampling protocol in the citizen-science eBird data, locational inaccuracies may also be a factor for the species’ presence data. To reduce the effects of potential locational or mapping error in the LULC and presence data, LULC covariates used in the model were “neighborhood” measures of abundance for a given LULC class, rather than per-pixel measures. Use of a neighborhood LULC measure provided not only site-level habitat information, but also provided information on habitat in the surrounding area. Individual species have unique, scale-dependent responses to landscape structure [35], [36], [37]. In modeling one individual species, it would be preferable to identify the appropriate scale of analysis that captures that species’ habitat preferences. However, the objective here was to identify relative influences of climate and LULC across 50 different species. Optimizing (varying) the scale of analysis for each individual species introduces another (unwanted) variable into the assessment. One set scale was thus selected to minimize the scale-dependent impacts on modeling results. In tests of multiple landscape scales for SDMs, Cunningham and Johnson [35] found that scales between 800 m and 1600 m were the most suitable a majority of 19 bird species tested. For this study, a 5×5 pixel (1,250 m×1,250 m) window around each point was chosen within which counts were tallied for each LULC class. The neighborhood counts for each LULC variable served as the LULC covariates within the modeling framework. A “LULC diversity” measure was also calculated, tallying the number of different LULC classes within each 5×5 window. The LULC diversity measure was also used as a covariate, as a measure of local landscape heterogeneity. **Table 3** summarizes the LULC covariates (as well as climate and topographic covariates described below).

#### Climate Data

The goal of this work was to examine long-term trends in bird species distributions in response to climate and LULC change. Global circulation models often produce climate data with monthly and yearly summaries, with year-to-year variability inherent in the output data. However, the use of average conditions was preferable to modeling with a single year of future climate projections, to minimize annual variability and focus on long-term trends [39]. A suite of global circulation models (GCMs) was used to obtain 30-year averages of climate consistent with IPCC SRES characteristics. A downscaling methodology similar to Hay et al. [39] was used to downscale coarse-scale climate data to a 4-km resolution for the conterminous United States [40], with data ultimately resampled to 250 m to match other covariates. Downscaled output was produced for six GCMs (BCCR-BCM2, CCSM3, CSIRO3.0-Mk, CSIRO-Mk3.5, INM-CM3.0, and MIROC 3.2) that provided climate data consistent with the IPCC SRES storylines (see http://www.ipcc-data.org/gcm/montly/SRES_AR4/index.html). Monthly data on average temperature, minimum temperature, maximum temperature, and precipitation were produced from each of the models. Variable averages across the six GCMs were calculated to reduce the bias present in any one individual model.

Covariates available for use in this assessment included not only yearly averages for temperature and precipitation, but also monthly averages, and monthly and annual minimum and maximum temperatures. However, not all variables were used, in order to reduce potential effects of multicollinearity. Correlation between potential climate variables was very high, particularly between the various temperature variables (Pearson correlation coefficient *r*>0.90 between nearly all paired temperature variables, such as monthly temperature and annual temperature). To minimize multicollinearity effects and to simplify data analysis, only the 30-year climate averages of annual temperature and annual precipitation, averaged across the six GCMs, were used as climate covariates in this assessment.

Bradley et al. [41] noted that the use of LULC data in conjunction with climate variables often does little to improve SDM results, due to collinearity of LULC and climate data at regional scales. There was little evidence of highly correlated LULC and climate variables in this assessment. Pearson correlation coefficients were computed for all LULC and climate covariate pairs. The highest correlation was between precipitation and the shrubland count (|*r|* = 0.39), while no other LULC and climate variable pair had |*r|* values higher than 0.29.

#### Topographic Data

The few studies that have used projected LULC data in conjunction with projected climate data to look at future species distributions have often restricted themselves to those two categories of data [7], [8], [16]. However, when developing SDMs for current conditions, modelers tend to use a wider array of input variables, with topography often playing a key role [1], [42], [43], [44]. Because the objective of this study was to assess the relative impacts of climate and LULC in “real-world” modeling applications, topography variables were included as covariates in this assessment in recognition that SDMs often do not focus solely on LULC and climate. Three topographic variables were used, based on the USGS National Elevation Dataset for the conterminous United States [45]; 1) elevation, 2) slope, and 3) compound topographic index (a measure of “wetness” and high flow accumulation). Each variable was resampled to match the geographic extent and 250 m spatial resolution of the LULC and climate covariates.

### Methods

#### Maximum Entropy Modeling Framework

MaxEnt model [46] (Version 3.3.1) running on a Windows desktop was used to model bird species distributions. Maxent was designed to model species distributions based on presence-only species data [26]. Maxent statistically minimizes entropy between the probability density of “presence” data, and probability density from “background” data, as defined in covariate space [26]. Maxent has been shown to be one of the most effective methodologies for modeling species distributions when presence-only data are used [2], [26].

Maxent estimates suitability for a given species by fitting feature classes based on environmental covariates. The filtered eBird data for each of the 50 species served as presence points. Environmental covariates were the LULC, climate, and topographic variables described above and shown in **Table 3**. Modeled feature classes in Maxent potentially included linear, quadratic, product, hinge, threshold, and categorical [46]. Linear features model linear response to a covariate, while quadratic features model response to the variable squared. Product features model interactions between paired variables. Hinge features model piecewise constant responses, while threshold features model abrupt boundary relationships between covariates and response. Category features are binary indicators used to indicate positive or null response to each class within a categorical covariate (e.g., thematic land cover map). All variables in this assessment were presented as continuous variables, including nominally thematic LULC data that were represented as counts within a 5×5 neighborhood around each point. Categorical features were thus not used in this assessment, but the other five Maxent features were used in modeling species response to the covariates.

The most widespread method for testing model results is a random hold-out of sample data [47]; 75% of the filtered eBird samples were used for training the model while 25% were reserved for testing. Maxent uses “background” points as locations where presence was not recorded, with background points either selected at random from the geographic extent of the study area, or specifically provided by the model user. The relationship between presence and background points in Maxent can strongly influence model results. Spatial bias in the presence points can result in a selection of background points with a fundamentally different spatial distribution [27], resulting in a model that represents the sampling effort as much as species presence [48]. A number of options were available to correct for spatial bias issues [25], [46], [49]. Several studies have discussed the use of spatially filtering or discarding records in over-sampled efforts [27], [48], [50], the approach used here and described above, with Kramer-Schadt et al. [26] finding it better reduced both errors of commission and of omission compared to other methodologies. Because the eBird data were spatially filtered, no attempts were made to account for bias through other measures.

Choice of the study area extent also can influence Maxent results [51], [52]. VanDerWal et al. [53] found that model performance suffered when background points were selected from either too restricted or too broad a geographic extent, in relationship to the presence points. Specifically, if background points are selected from too broad a geographic area, predictive models were dominated by coarse-scale determinants of distribution (such as climate) [53], while those that use too limited a geographic area underestimate the importance of these variables [52] To reduce the influence of a mismatch between background area and “presence” points, a consistent buffer was applied around 2001 (contemporary) input presence points to construct a unique geographic extent for each species. The buffer zone was used to definitively set the study area for each species, both for defining where background points could be selected by Maxent, and to set the complete geographic range for modeling both current and future distributions. Ideally a unique geographic region would be optimized for each species according to characteristics of the observation data [51], but to facilitate comparison across the 50 species, a consistent buffered extent was used for all species. VanDerWal et al. [53] used a 200-km buffer, but initial experimentation for this assessment found that to be too restrictive for changes in conterminous United States bird species range from 2001 to 2075, with some species’ ranges shifting by more than 200 km. A 500-km buffer around input eBird points was used, restricting both the range from which background points could be selected, and restricting the prediction space for each species’ range.

Remaining parameterization of Maxent largely followed model defaults. Anderson and Gonzalez [54] and Warren and Seifert [55] recommended species-specific tuning of Maxent settings, noting that the regularization value (used to restrict model “over-fitting” to input data) had a large effect on results. However, Phillips and Dudik [46] tested regularization values and found that “regularization parameters which are the defaults in MaxEnt software…are well suited for a wide range of presence-only datasets.” The six feature types are also selectable, yet Syfert et al. [56] found little influence on model results by varying the feature types that are used. Phillips and Dudik [46] found that using the default 10,000 background points achieved similar model results as if all possible background sites were used; the default setting was thus used. Default settings were also used that enabled “clamping” of covariate and feature values for the 2075 model simulations. With the model trained on the 2001 covariate data, the potential existed for “novel” covariate values when the model was applied in 2075, using projected climate and LULC data. For the 2075 model simulations, the enabled clamping resulted in a rescaling of both covariate and feature values if their values were higher or lower than those found in the training data. Values higher than those encountered in the training data were rescaled to the training data maximum, while values lower than those encountered in the training data were rescaled to the training data minimum. The implications of the use of clamping are provided in the discussion section.

Parameterizing Maxent as described above, initial model simulations for each species were conducted using the filtered eBird data for presence points, and the 2001 LULC, 2001 climate, and topographic variables as covariates. Twelve model simulations were made in total for each species (**Table 4**). A base model simulation was done for 2001 using all variables (simulation 1), while additional simulations were done for 2001 with climate and topography (excluding LULC) (simulation 2) or land cover and topography (excluding climate) (simulation 3). The model developed for simulation 1 was applied for 2075 to examine potential future impacts of climate change, LULC change, or both (topographic variables are static in all simulations). For each of the three IPCC scenarios, simulations were done with all 2075 variables, with projected climate but static LULC, and with projected LULC but static climate. Keeping either climate or LULC static from 2001 to 2075 allowed for the examination of the relative effects of projected climate versus projected land use change on future bird species distributions. Three 2001 simulations and nine 2075 simulations were thus conducted for each of the 50 species, resulting in 600 individual model simulations.

**Table 4:** Twelve model simulations were conducted for each species, three for 2001 and nine for 2075.

Simulation	Description	Climate (Scenario)	LULC (Scenario)	Topo Data	Scenario
**1**	2001 All	2001	2001	Yes	-
**2**	2001 Climate	2001	-	Yes	-
**3**	2001 LULC	-	2001	Yes	-
**4** *	2075 A1B All	2075 A1B	2075 A1B	Yes	A1B
**5** *	2075 A1B Climate Change	2075 A1B	2001	Yes	A1B
**6** *	2075 A1B LULC Change	2001	2075 A1B	Yes	A1B
**7** *	2075 A2 All	2075 A2	2075 A2	Yes	A2
**8** *	2075 A2 Climate Change	2075 A2	2001	Yes	A2
**9** *	2075 A2 LULC Change	2001	2075 A2	Yes	A2
**10** *	2075 B1 All	2075 B1	2075 B1	Yes	B1
**11** *	2075 B1 Climate Change	2075 B1	2001	Yes	B1
**12** *	2075 B1 LULC Change	2001	2075 B1	Yes	B1

Model simulations variously include or exclude climate and LULC covariates in order to assess the individual effects of each.

*Simulations for 2075 used the model developed for run 1 (2001 “All”), applying 2075 climate and/or LULC data from the appropriate scenario.

#### Assessing Model Results

Several different metrics were used to assess the relative impacts of climate and LULC change on bird species distributions. The three 2001 model simulations were assessed for model fit through a comparison of Area Under the Curve (AUC) of the Receiver Operating Characteristic (ROC). AUC values represent the probability that a randomly selected “presence” site will have a higher AUC value than a randomly chosen “background” site. Comparison of AUC scores was used to examine relative impacts on model fit when LULC or climate data were excluded from the analysis. A second criterion was the relative contributions of the covariates to model results, measured by relative changes in regularized training gain between variables. This information was provided as a “percent contribution” from Maxent. A third criterion was a comparison of modeled “suitable” range for each species. Elith et al. [26] cautions against cross-species comparisons using logistic output from Maxent, as probability of presence is relative to the sampling effort for a given species. However, changes in relative range for each individual species can be identified by applying a threshold value to Maxent’s logistic output, to differentiate between likely presence and absence locations. The “maximum sensitivity plus specificity” threshold was used [15], [38], a thresholding technique that limits both errors of commission and errors of omission and has been found to outperform other techniques [57], [58].

The 2075 simulations were evaluated by assessing changes in “suitable” breeding range as compared to 2001. Net change in range area was determined for each species by first applying the “maximum sensitivity plus specificity” threshold to modeled output and then differencing the threshold results, with comparisons of net effects of climate change alone, LULC change alone, and both climate and LULC change from 2001 to 2075 (for each scenario).

Finally, results were examined in terms of species range and relationship to the conterminous United States study area. While data sources and analyses often stop at political boundaries, species ranges obviously do not, and the use of conterminous United States borders for this work resulted in the modeling of truncated ranges for many species. Both 2001 and 2075 model results could be impacted dependent upon whether the entire range was modeled or if one or more maximum extent boundaries were artificially truncated [51], [52], [53]. Many SDM applications model truncated species distributions (see discussion below); assessing results on species range characteristics allowed for an examination of LULC and climate impacts across a variety of “real-world” modeling situations. For each of the assessment criteria discussed above, mean values were provided (**Table 5**) for species within the following “range classes”: 1) “Single Truncated” (species with either the northern *or* southern extent artificially truncated by United States borders, 2) “Double Truncated” (species with ranges truncated at *both* the northern and southern United States border, and 3) “Whole Ranges” (species with >95% of current breeding ranges found within the conterminous United States, measured with NatureServe species distributions [24].

**Table 5:** Impacts on assessment variables by range class.

	Single Truncated	Double Truncated	Whole Range
**5(A) –2001 MODEL FIT (AUC Score – Mean Value)**			
**All Variables**	0.916	0.839	0.906
**LULC, No Climate**	0.891	0.834	0.892
**Climate, No LULC**	0.891	0.799	0.888
**5(B) –2001 VARIABLE CONTRIBUTION (in percent)**			
**Climate Variables**	49.5%	52.8%	52.7%
**Topography Variables**	12.8%	6.0%	13.9%
**Land Cover Variables**	37.7%	41.2%	33.4%
**5(C) –2001 Range (Mean Values – Percent of conterminous United States area)**			
**All Variables**	23.0%	42.2%	26.6%
**LULC, No Climate**	28.4%	43.7%	30.1%
**Climate, No LULC**	31.1%	57.0%	32.8%
**5(D) –2075 Breeding Range (Mean Values – Percent change from 2001)**			
**All Variables**	−9.9%	+2.6%	+12.0%
**LULC, No Climate**	+3.8%	+3.5%	+1.5%
**Climate, No LULC**	−13.0%	+1.2%	+10.2%

Values represent mean values across all species in a class. “Single Truncated” (27 species) represents species with ranges artificially truncated at either the north *or* south by the United States border. “Double Truncated” (15 species) represents species with truncated ranges that extend to or past the United States/Canada border in the north and the United States/Mexico border in the south. “Whole Range” (8 species) represents species where >95% of the current range is found within the conterminous United States.

## Results

### 2001 Models (“current” species’ distributions)

**Figure 1:**
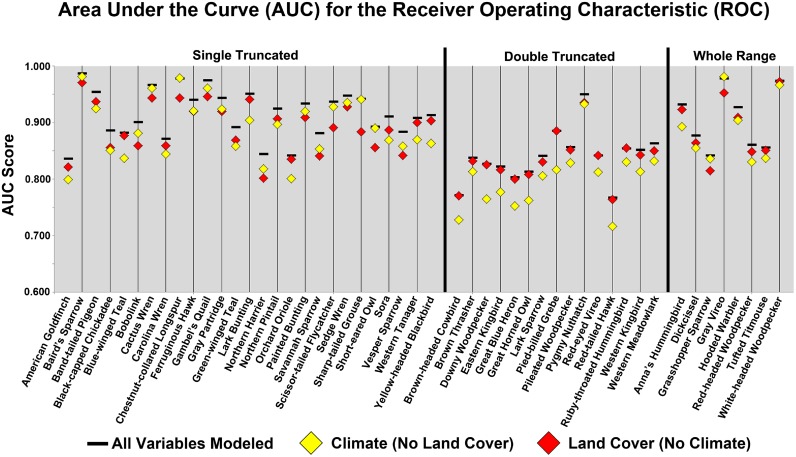
AUC scores for each species, for run 1 (all variables modeled), run 2 (Climate, no Land Cover), and run 3 (Land Cover no Climate). AUC scores are also parsed by range class.

The 2001 models were assessed for model fit using AUC scores. Values of 0.5 indicate model fit was no better than random, while increasing values above 0.5 indicated an improved model fit. **Figure 1** provides AUC scores for the three 2001 model simulations for each of the 50 species. AUC scores ranged from a low of 0.716 to a high of 0.987, with considerable variation among species, as well as among the three model simulations for a given species. Model simulations with all variables included (simuilation 1) had the highest mean AUC score, at 0.891, and the highest AUC score for each of the 50 species. AUC scores were significantly lower (p<0.001; paired t-test) for both simulation 2 (climate, no LULC) and simulation 3 (LULC, no climate), with mean AUC scores of 0.863 and 0.874, respectively. Results indicate significantly poorer model fit when LULC data were excluded than if climate data were excluded (p<0.01; paired t-test). By range class AUC scores were significantly lower when either LULC or climate data was omitted, for all range classes (p<0.01, paired t-test) (**Table 5A**). AUC scores overall were similar for the Single Truncated and Whole Range classes, but were much lower on average for the Double-Truncated class. Omission of LULC resulted in the lowest overall AUC score for every species in this class. The relative impact of LULC or climate data omission was more balanced for the other two range classes and varied by species.

**Figure 2:**
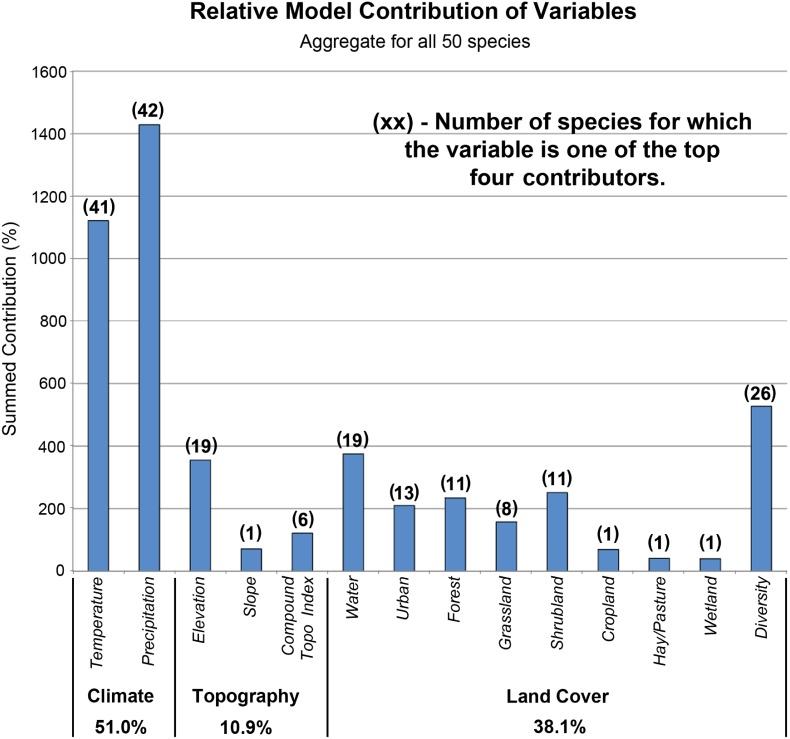
Proportional contributions of each covariate to the regularized training gain, aggregated across all 50 species.

**Figure 2** depicts Maxent-provided proportional contributions of each covariate to the regularized training gain, aggregated across all 50 species for simulation 1 (all variables modeled). The climate covariates played an important role in shaping 2001 simulations, with annual temperature and precipitation providing 51.0% of the contribution to model results. Temperature was one of the top four contributing covariates for 41 species, while precipitation was one of the top three covariates for 42 species. LULC variables in aggregate contributed 38.1% to model results, while topographic variables contributed 10.9%. Results vary among individual species, but overall, it is clear that both climate and LULC were important contributors to model output when both were included as covariates. Results were similar when categorizing species by range class (**Table 5B**).

**Figure 3:**
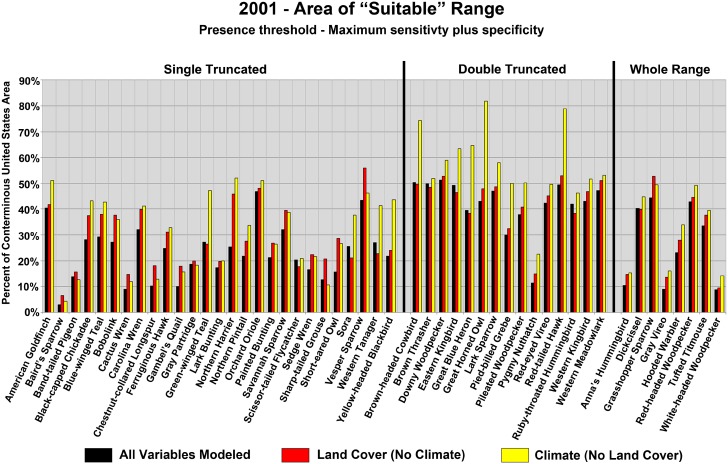
Area (percent of conterminous U.S. land mass) classified as suitable to support a given species, for model run 1 (all variables modeled), run 2 (Climate, no Land Cover), and run 3 (Land Cover no Climate). Suitability was determined by applying the maximum sensitivity plus specificity threshold to Maxent logistic output. Results are also parsed by range class.

**Figure 3** provides a comparison of modeled “suitable” area for each species, among model simulations 1, 2, and 3, using the unique maximum sensitivity plus specificity threshold criteria for each species and simulation. Values are presented as a percentage of the total land surface for the conterminous United States with Maxent logistic output values above the threshold criterion. While the predicted suitable range for a given species was sometimes similar across each of the three 2001 model simulations for a species, in many cases, the area deemed to be suitable varied dramatically depending upon what variables were used as covariates. For 36 of the 50 species, the area deemed suitable was highest in simulation 2, when only climate and topographic variables were used as covariates (LULC excluded). The area deemed suitable was nearly double in some cases (e.g., Great Horned Owl (*Bubo virginianus*), Yellow-headed Blackbird (*Xanthocephalus xanthocephalus*)) for simulation 2, as opposed to simulation1 when LULC data were also incorporated. For the other 14 species, the area deemed suitable was highest for simulation 3, when only LULC and topographic variables were used as covariates (climate excluded). Adding covariates to the model, be they LULC or climate, clearly acted to further define (and restrict) the area deemed to be suitable for species’ habitation. Using climate data alone resulted in broad, overly generalized suitability ranges if LULC data were not used to help further define suitable landscapes. Results were similar when evaluating the three different range classes (**Table 5C**), with the smallest range consistently modeled when all variables were used as covariates. However, for the Double Truncated range class, the omission of LULC data from the model resulted in a much larger increase in range as compared to the other two range classes, while omitting climate data had little impact.

### 2075 Models (“projected” species’ distributions)

**Figure 4:**
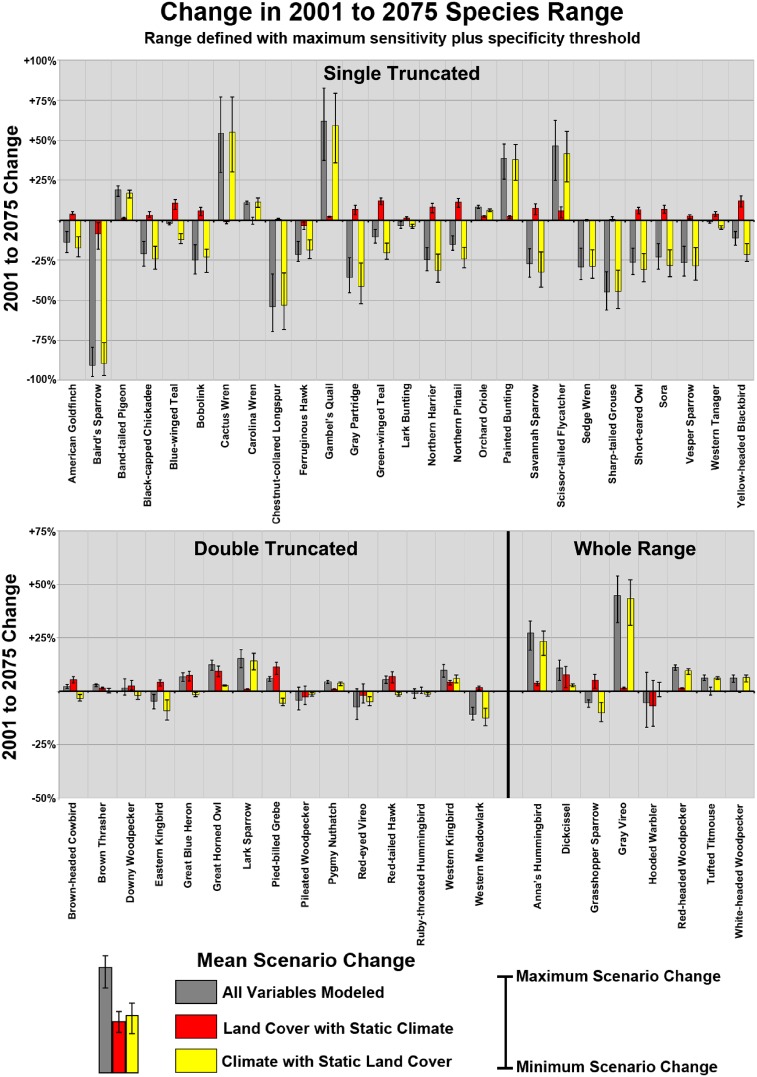
Changes (2001 to 2075) in area classified as suitable to support a given species. Change is presented as area change, relative to the contemporary (2001) modeled range. Bar height represents mean change across the 3 IPCC scenarios, while error bars represent scenario variability. Suitability was determined by applying the maximum sensitivity plus specificity threshold to Maxent logistic output. Results are also parsed by range class.

**Figure 4** depicts projected changes in range for each of the 50 species, measured as change relative to the range modeled in 2001 (simulation 1), using the maximum sensitivity plus specificity threshold to differentiate between presence and absence. Range differences are provided for each of the 3 model simulations, for each of the 3 scenarios, with bar height providing the mean change in range across all three scenarios, and deviation bars providing the variation between scenarios. Depending upon species, modeled changes in range varied according to which covariates were used and between different IPCC scenarios. Changes in range varied from a near complete loss of all conterminous United States suitable range (Baird’s Sparrow(*Ammodramus bairdii*)) to range expansions that nearly double the current range (Cactus Wren (*Campylorhynchus brunneicapillus*), Gambel’s Quail (*Callipepla gambelii*), Gray Vireo (*Vireo vicinior*)). **Figure 4** indicates that the magnitude of projected changes in range was much more strongly impacted by projected climate change than by projected LULC change, when using a threshold to define suitability. When only LULC changed (climate static) from 2001 to 2075, changes in projected ranges from 2001 were highly significant (*p*<0.001; paired t-test) but were never more than 20% (either positive or negative). When only climate changed (LULC static) from 2001 to 2075, range changes were often quite dramatic, with 20 species showing range changes of 25% or more for a given scenario. Climate and LULC could either both influence species’ distributions in the same direction, or a positive species response to one category of covariates could be offset by a negative species response to the other category.

**Table 5(D)** and Figure 4 show substantial differences in the relative effects of LULC and climate on 2075 model results, depending upon range class. The most dramatic overall changes in range were in the Single Truncated class, where climate change obviously had a strong effect on model results. Climate change had a much more muted impact on the Double Truncated class, with low overall changes in range. Climate had moderate to strong impacts for the Whole Range class. The impacts of LULC change were much more consistent across range classes than were the impacts of climate change.

### Species Focus - Hooded Warbler (*Wilsonia citrina*)

**Figure 5:**
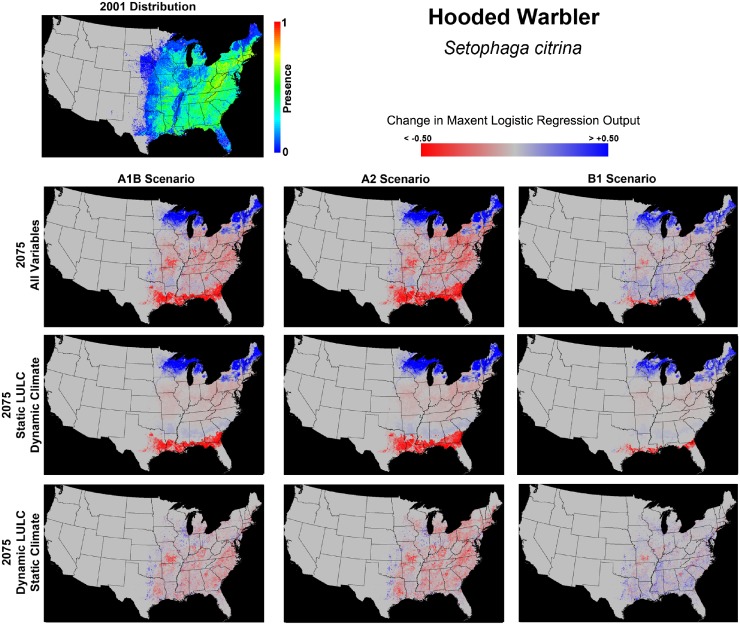
Maxent 2001 logistic output for the Hooded Warbler, and projected changes under each 2075 scenario and model run. Climate change results in broad northward shifts in species range across all scenarios. LULC change alters the local pattern of habitat suitability, with losses under the A1B and A2 scenarios, and general increases in the B1 scenario.

While it is impractical to individually discuss each of the 50 modeled species, the relative impacts of climate and LULC change on one species, the Hooded Warbler (*Wilsonia citrina*), are highlighted here to demonstrate specific impacts of climate and LULC. The Hooded Warbler is a forest-dependent species that primarily breeds in the eastern United States. **Figure 5** provides 1) a map of Maxent logistic output for 2001, using simulation 1 (all covariates modeled), and 2) changes in output for each 2075 scenario, and for each 2075 model simulation. The AUC score for simulation 1 (2001) indicated a high-level of model fit (AUC = 0.927), with precipitation, temperature, and forest count (in relative order) measured as the three covariates contributing the most to model results. For simulation 1, 23.1% of the conterminous United States was deemed “suitable” (threshold) range for the Hooded Warbler. The predicted range sharply increased to 27.9% in simulation 3 (climate excluded) and 33.9% for simulation 2 (LULC excluded), a pattern seen for many species (**Figure 3**).

Changes in predicted range by 2075 indicate a strong influence of both climate and LULC change (**Figure 5**). The economically focused A1B and A2 scenarios are similar, as a changing climate resulted in strong shifts in overall species range, with large contiguous bands of losses of range in the south and gains in the north. The effects of LULC change are more fragmented, but substantial forest loss results in local areas of decline throughout much of the eastern United States. The effects of climate change are more muted for the environmentally focused B1 scenario, with less severe shifts to the north. While local areas of forest loss do result in range declines in the B1 scenario, afforestation and forest regeneration result in higher presence scores in many locations.

**Figure 6:**
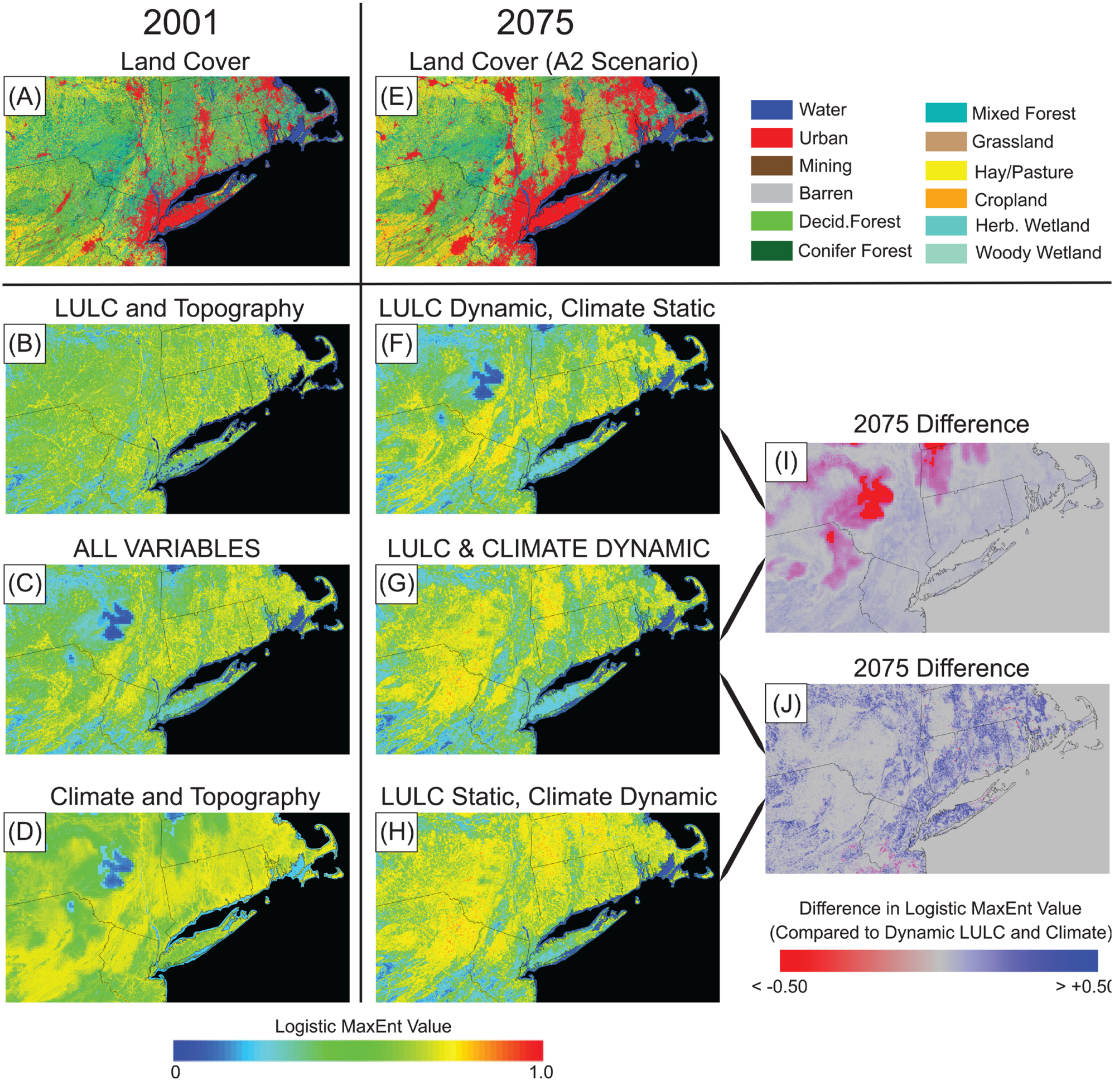
LULC and model results for a portion of the Hooded Warbler’s range. Panels on the left (6 a–d) depict 2001 LULC, and the three model runs for 2001. Panels in the middle (6 e–h) depict 2075 LULC for the A2 scenario, and the three model runs for 2075 for that scenario. The two panels on the right depict differences in model results (compared to 6 g, when all variables were modeled) if 1) climate was held static for 2075 (6i), and 2) LULC was held static for 2075 (6j).

**Figure 6** displays modeling results for the Hooded Warbler for both 2001 and 2075 (A2 scenario) for a smaller area within their current breeding range. At this scale the relative impacts of both LULC and climate are evident for both current (2001) modeling, and for future projections. **Figure 6(a)** and **6(e)** show LULC change from 2001 to 2075, characterized by substantial expansion of urban and agricultural lands, at the expense of forest land. **Figures 6(b)**, **6(c)**, and **6(d)** show model results with LULC and topography as covariates, all variables as covariates (LULC, climate, and topography), and climate and topography as covariates, respectively. Without the use of climate data, suitability was highly heterogeneous, but higher elevation areas that currently do not support Hooded Warbler populations (e.g., parts of the upper-left quadrant) often had high suitability values even with the use of topographic information (**Figure** **6(b)**). Without the use of LULC data, suitability was less heterogeneous and the cooler high-elevation areas were characterized by lower values, yet areas of dense anthropogenic land-use that are unsuitable for Hooded Warbler breeding were often characterized as highly suitable (**Figure 6(d)**). The use of both LULC and climate data, in conjunction with topographic data, resulted in a heterogeneous distribution of suitability values, capturing both the influence of cooler high-elevation areas as well as areas of dense anthropogenic land-use (**Figure 6(c)**).

For 2075 model simulations, **Figure 6(g)** shows the impacts on Hooded Warbler range when both projected climate and projected LULC are used in the model. Range expansion occurred towards higher elevations as a warming climate results in more suitable breeding conditions. LULC change, primarily urbanization and agricultural expansion, resulted in large but heterogeneous losses of breeding range, counter-balancing range gains due to climate change. **Figure 6(f)** shows a model simulation with projected 2075 LULC but a static 2001 climate. Without the use of projected climate data, the range expansion due to warming was not captured (**Figure 6(i)**). **Figure 6(h)** shows a model simulation with projected 2075 climate but a static 2001 LULC, where many climatically suitable areas were still noted as suitable for breeding despite the substantial loss of forest habitat (**Figure 6(j)**).

Specific results for all 50 species, including range maps as provided in Figure 6, are accessible through a companion website (http://landcover-modeling.cr.usgs.gov/sdm.php).

## Discussion

### Research Questions

**What are the relative influences of LULC and climate in modeling contemporary (2001) breeding bird distributions?** Clearly both climate and LULC change impact current bird species distributions, with relative impacts that are species specific. 2001 model fit was generally better with LULC simulations (climate excluded) than for climate simulations (LULC excluded), yet climate data covariates contributed more to model results than LULC data. One story that arises from these seemingly conflicting results is one of scale. Results suggest that climate data alone, without constraints afforded by the use of habitat (LULC) data, provide a “broad-brush” picture of suitability for a given species. LULC data alone excel at providing local-level insight to site-level habitat suitability. Given the inherent heterogeneity of the moderate-scale LULC data used here compared to variations in climate across geographic space, it is not surprising that climate alone offers only a general characterization of species range. For the 2001 species models, the modeled area deemed to be suitable to support a species was generally much higher when climate data were used without LULC data (**Figure 3**). The addition of LULC data to climate-based model simulations greatly restricted modeled species ranges in most cases. Prince et al. [59] similarly described climate as determining overall potential carrying capacity for a species, but noted the impact of climate change itself may be overestimated, as other factors that determine local suitability must be assessed. These results suggest that the use of climate data, without supporting LULC data, likely results in errors of commission, where climatically suitable regions are labeled as appropriate for supporting a given species, despite underlying LULC conditions that make actual presence unlikely. Araujo and Peterson [9] discussed such commission errors in bioclimatic envelope modeling, attributing overzealous predictions of range to an incomplete model; in this case, climate-only models for 2001 are “incomplete” without supporting LULC data.

Actual species range in relationship to the modeled study area influenced 2001 model results. Model fit was negatively impacted if the study area was largely contained within the actual species range. This was the case for the Double Truncated species, where ranges spanned all latitudes in the conterminous United States and were truncated at both the northern and southern borders. Climate data, in particular temperature data, thus did little to improve model fit, as species occurrences already spanned most potential climate regimes within the conterminous United States. With the resultant small impact of climate data, overall model fit suffered (**Figure 1** **;** **Table 5a**) and the addition of climate data did little to improve model results over the LULC and topography model (**Figure 1**). Modeled species range was also influenced by the relationship between actual range and study area. As noted above, the use of climate data without LULC data often resulted in errors of commission, but these errors were magnified for the Double Truncated species, with an over-prediction in suitable range as compared to the complete model with both climate and LULC data (**Figure 3**).

**What are the relative impacts of projected climate and projected LULC change on breeding bird distributions?** For modeled species ranges, projected changes in climate provided more dramatic shifts in future species’ ranges than did projected LULC change. LULC change alone altered suitable range by no more than 20% for any species, yet climate change resulted in shifts of 50% or more for several species. Differences between the three different scenarios were often substantial, with some scenarios projecting double the range shift compared to other scenarios. However, the overall storyline was climate change impacting net changes in species range more than projected LULC change.

The relationship of the actual species range to the study area obviously affected 2075 results, with climate impacts often over- or under-estimated in relationship to LULC impacts, depending upon species. The Single Truncated class contained species where either the northern or southern extent of their actual range was artificially truncated by the borders of the study area. With a warming climate, for species with ranges truncated along their southern extent but not the north, the models thus predicted overall range expansion to the north, without capturing the (presumed) range contraction due to climate change at the species’ southern range extent. Conversely, for species with ranges truncated along their northern extent but not the south, the models predicted overall range contraction, capturing contraction in the south but failing to capture (presumed) expansion in the north. By only capturing “half of the story” (i.e., either capturing range expansion in the north or range contraction in the south), these results provided an unrealistically high impacts of climate on *net* range, either positive or negative. While the impacts of climate on *net* change were thus likely overestimated for these species, *gross* change was likely underestimated, as half of the story was “missing”. For the Double-Truncated class, the relative impacts of climate change to LULC change were likely underestimated. For these species, the impact of climate on species range was artificially dampened by the truncation of northern and southern range boundaries, areas where range could potentially expand or contract, respectively, due to a warming climate. For the Whole Range species, the relative impacts of climate change versus land use change vary, with net change values only providing part of the story as evidenced when assessing results for the focus species, the Hooded Warbler.

**What are the specific impacts of climate and LULC on one focus species, the Hooded Warbler?** The presented results for the Hooded Warbler mirrored those for many of the 50 modeled species. Net change in breeding range area showed relatively little change, while geographic patterns change dramatically. Climate change resulted in a broad overall shift in range to the north and to higher elevations, while LULC change resulted in heterogeneous, local-scale changes in habitat suitability. The breeding distributions of the Hooded Warbler have been found to be highly correlated with climate variables [60]. The species was unknown as a breeder in Canada until 1949, but with a warming climate they have started to breed in increasing numbers in extreme southern Ontario [60]. Melles et al. [60] modeled the relationship between climate and habitat covariates and the Hooded Warbler range, and found strong relationships between range expansion to the north and changes in climate over the last few decades, with habitat availability acting as a constraint on expansion. Naujokaitis-Lewis et al. [61] examined the potential impacts of climate change out to 2080 on Hooded Warblers and projected breeding range shifts to the north with characteristics dependent upon which GCM was assessed. However, they also found that land-use pressures around the Great Lakes were limiting factors to range expansion, and recommended future work that focused on “the development of more realistic (habitat) loss scenarios”. The newly available LULC projections used in this work allowed for such an analysis.

As discussed, most future projections use projected climate data but ignore future LULC change. **Figure 6** clearly indicates that for a species such as the Hooded Warbler where climate change drives a broad overall shift in range to the north and to higher elevations, the modeled extent of suitable range at a local level can potentially be misrepresented without the use of projected LULC data. In this case, habitat loss due to urbanization and agricultural expansion would be missed without the use of LULC data, resulting in an over-prediction of suitable range (**Figure 6j**). Alternatively, without the use of projected LULC data, suitable range may be under-predicted if beneficial LULC change occurs (e.g., Grasshopper Sparrow (*Ammodramus savannarum*) results within the Eastern United States, where projected clearing of forest land in most scenarios resulted in more suitable habitat conditions by 2075). Exclusion of climate data can also result in a misrepresentation of modeled range. For the Hooded Warbler, range expansion to higher elevations was missed for the 2075 model excluding climate data (**Figure 6i**).

**What are the implications for the use of climate and LULC data in SDMs?** For contemporary species modeling or for projected changes in species range, both climate and LULC data should ideally be used. In general, model fit consistently increases with the use of both climate and LULC data, while predicted suitable range decreases. The implication is that information is missing from SDMs if both climate and LULC are not used as covariates. For modeling of current species range, areal summaries of modeled range (**Figure 3**) as well as spatially explicit maps of modeled range (**Figure 6b, c, and d**) show that SDMs relying on climate data without LULC data provide only a broad-brush, generalized species range, while LULC data alone provide site-level information on habitat suitability while omitting climatic thresholds of unsuitability. Dependent on application, a broad-brush generalization of a species range may be adequate. However, it should be recognized that the results likely over-represent the area of suitable range and fine-scale detail is unlikely to be obtained.

Exclusion of LULC data is primarily an issue for projections of future species’ range. As shown here, bioclimatic modeling where LULC information is not included or is considered static likely results in a misrepresentation of future species’ range. For example, Hooded Warbler results provided here and in past studies indicate that while climate drives broad-scale shifts in range, SDMs likely misrepresent the extent of future range shifts if LULC change is not taken into account. Given how little projected LULC data is used in modeling future species distributions, quantitative estimates of range shifts are likely overestimated if habitat loss dominates projections of LULC change, or underestimated if habitat gain dominates projections of LULC change. Bioclimatic models that do not use any form of LULC information, even static LULC information for the future, likely overestimate suitable ranges (**Figure 3**).

The relationship between the study area and the actual species range also needs to be strongly considered, both in the project design and assessment phases. The methodology used here mimics that of many modeling applications. Of all the citations included in this paper where species distribution and/or probability-of-occurrence modeling was done, over two-thirds (21 of 31) of model applications assessed only partial/truncated species ranges. Many of the recommendations referenced in this paper with regard to model parameterization and handling scale issues [35], [38], [46], [53], spatial bias and other issues with presence data [23], [48], [50], and relative influences of climate and LULC [1], [8], [12], [16], [59], [60] were derived from studies where only partial ranges were assessed. Despite the prevalence of modeling of partial ranges, the results here indicate that caution is needed in project design, both for accurate modeling of species range, and for the interpretation of modeling results. Modeling of an entire species’ range may improve model fit and enable a more direct interpretation of results, yet is often not practical due to data or processing limitations. While modeling a partial range is thus unavoidable in many cases, model results should be interpreted within the context of the overall project design and the relationship between species range and the study area. Modeling results may still be “valid” when using truncated ranges, but if the intent is to study the impacts of climate change on species distribution, for example, then the use of a “double-truncated” study area boundary would obviously be a poor choice, as the effects of climate would likely be artificially muted. If the intent is to quantify specific impacts of LULC and climate, disentangling the relative effects of LULC, climate, and other covariates would be complicated by the modeling of truncated ranges.

### Comparison to Existing Research

The conceptual approach behind Barbet-Massin et al. [7] modeling of bird species in Europe and Matthews et al. [1] modeling of eastern U.S. bird species are likely the most similar work to this assessment. Each assessed a large number of species across broad geographic regions, and both incorporated projected climate and projected LULC data. Similar to Barbet-Massin et al. [7], this assessment found that LULC-based models alone predicted smaller overall shifts in future range size than did climate-based models. However, these results differ from multiple studies that discussed the relative influence of climate versus LULC, including components of Barbet-Massin et al. [7]. Barbet-Massin et al. [7] found that modeling accuracy was higher with climate-only variables than with habitat-only variables; in this study, the opposite was true in the majority of species that were assessed. Thuiller et al. [12] found that the inclusion of LULC covariates improved explanatory power of bioclimatic models, but that the “addition of land cover variables to pure bioclimatic models does not improve their predictive accuracy”. In this assessment, on average, AUC scores declined more in the absence of LULC data than in the absence of climate data, while for all 50 species, 2001 model fit was improved when LULC data were included as a covariate as opposed to models with only climate and topographic data.

The differences in results may potentially be explained by 1) the difference in scale between the different assessments, 2) variations in the number of climate covariates, and 3) the use of topographic data within this assessment. Barbet-Massin et al. [7] used much coarser, 0.5-degree resolution LULC data, and noted that “such a resolution was probably too rough to precisely account for habitat factors.” Thuiller et al. [12] also used a very coarse spatial resolution (50-km grid cells) and noted results may differ at finer resolutions. Bucklin et al. [13] similarly found that LULC variables provided little benefit in SDMs, but noted that both thematic and spatial resolution improvements over their LULC data source may have provided different results. Barbet-Massin et al. [7] and Thuiller et al. [12] also noted the lack of a measure of fragmentation or landscape heterogeneity in their assessments; in this study a LULC diversity measure was used to represent heterogeneity. This assessment also used only two climate covariates, while Barbet-Massin et al. [7], Thuiller et al. [12], and Bucklin et al. [13] each used eight climate covariates. Additional research is needed to assess the optimum combination of covariates and how covariate choice impacts results, particularly for heavily correlated climate covariates. The use of topographic data in this assessment also may have impacted the relative impacts of LULC and climate data. The information content provided by topographic data alone, or topographic data in combination with LULC data (e.g., “product features” within Maxent that assess 2-way interactions between covariate pairs) may partially mimic or replace the information content that is provided by climate variables [42].

Matthews et al. [1] also modeled U.S. bird species, and also used projected climate data, projected LULC data, and topographic data to assess future changes in distributions. They modeled the eastern portion of the United States at a very coarse spatial resolution (20-km grid cells), with changes in tree species representing the only modeled form of LULC change. Similar to their results, the predictive power (indicated by goodness-of-fit measures) of the models described here decreased when only climate and elevation data were used as predictor variables (i.e., LULC excluded). Even with the differences in spatial resolution, both studies found that modeling with only climate and topographic variables leads to generalized species distribution maps that lack fine-scale detail. Matthews et al. [1] noted that modeling with climate and topographic data alone makes the resultant models much more susceptible to over-prediction of future impacts of climate change on species range. It should also be noted that their use of the eastern United States as the study area resulted in artificial truncation of nearly all modeled species ranges.

### Caveats and Future Research

In assessing potential future changes in species ranges, caution has been recommended when attempting to apply a contemporary model to future climate conditions [26], [46]. Transferability of model results is confounded when novel conditions (i.e., specific combinations of covariates not found in the original model’s training data) are found for future dates or for other geographic regions. In this assessment novel conditions were most likely to occur with higher temperatures due to climate change. However, the use of the 500-km buffer to establish the study area for each species, as well as the use of the clamping feature in Maxent, resulted in a muted influence of novel conditions on model results. Selecting background points within a 500-km buffer of a species’ current range enabled the collection of background points with higher (points selected south of the breeding range) or lower (points selected north of the breeding range) average temperatures than those found in the breeding range. Thus temperature was often used as a threshold feature in species’ models, with conditions modeled as unsuitable if temperature in a given location was above or below a modeled tolerance level for the species. For example, the breeding range for the Bobolink (*Dolichonyx oryzivorus*) covers much of the northern United States, but they are absent in southern areas. An examination of the 2001 model developed for the Bobolink shows average temperature used as a threshold feature. For a species such as this, with the southern end of its breeding range currently within the conterminous United States, novel conditions potentially introduced by a warming climate were unimportant, as the model already ensured exclusion of the species as a breeder in areas with temperatures above threshold values found in the training data.

Novel conditions could potentially be a problem for species with current breeding ranges extending to the United States and Mexico border. No background data south of the border were used to train the model. With a warming climate, for some species, it is likely that local temperatures in the projected climate data exceeded any temperatures found in the training data. The clamping feature in Maxent was used to control novel conditions in situations such as this, effectively rescaling novel covariate values to maximum values found in the training data. Clamping thus eliminated statistical issues with applying models into a novel prediction space, but by rescaling extreme values in the projection space, the model may effectively be dampening the impact of future change on future species distributions for 2075. Clamping of novel temperatures, for example, could result in the model incorrectly representing far southern portions of a species range as “suitable” for breeding, when in fact a temperature tolerance limit has been reached and has pushed the southern limit of the breeding range north of the United States and Mexico border.

There are additional potential caveats in interpreting results of this assessment. These results are based on one modeling methodology (Maxent), with one defined method for parameterization. Many papers have focused on the effects of different parameterization settings when using the Maxent model [27], [48], [56], and it was not the intention of this paper to revisit how different parameterizations affect Maxent results. The results presented here were also conducted at one specific spatial scale, with one specific suite of covariates and bird presence data. It was impractical to perform comprehensive analyses across all possible permutations of modeling frameworks, parameterization settings, spatial scales, thematic scales, temporal resolution, and data sources; results may differ for assessments where these components are altered. There was no attempt to rigorously address all potential sources of modeling uncertainty in this assessment. Conlisk et al. [38] attempted to disentangle all sources of uncertainty in SDMs, concluding that the modeling framework itself is the most important source of uncertainty. Ideally multiple models would be used to also disentangle effects of the modeling frameworks themselves, but resources were unavailable for a multi-model assessment given the large number of species, and multiple combinations of dates, covariates, and scenarios. Other potential drawbacks to the approach used here is an oversimplified representation of the driving forces behind species distributions. One final area that needs further exploration is the correction of bias for eBird data. Spatial bias was mitigated by spatially filtering the data. However, given the number and diversity of species modeled, a consistent filtering threshold of 20 km was used for all species; no attempts were made to tailor the filtering protocol to the spatial data characteristics for each species, nor were attempts made to quantify the reduction in spatial bias in this assessment. Additional potential sources of error and bias in eBird data that were not accounted for include accuracy of geographic data entry and highly variable observation and identification skills among eBird participants [62], [63].

## Conclusion

This work represents the first assessment of the effects of climate and LULC for bird species in the conterminous United States using both 1) newly available LULC projections of high-spatial and thematic resolution and 2) climate and LULC projections that are both consistent with IPCC SRES scenario frameworks. While modeling results clearly indicate a species-dependent determination of the relative impacts of climate and LULC change on both current and future range, it is clear that SDMs benefit by including both climate and LULC covariates. The use of climate data alone likely results in errors of commission and an over-prediction of current range. For future modeling of species range, the use of climate change information without corresponding LULC change may result in the misrepresentation of future range either positively or negatively, dependent upon whether projected LULC change was harmful or beneficial to a species. The inclusion of LULC data in SDMs 1) significantly increased measures of model fit, and 2) “tempered” predicted ranges from climate-only modeling frameworks by providing fine-scale information on local habitat suitability. When modeling future shifts in range, climate had the dominant impact on range shifts, yet LULC change was dominant for many species. Relationship of the species’ range to the geographic bounds of the study area also clearly impacts whether climate or LULC has the dominant effect on modeled species range, and needs to be considered at both the design and assessment stages of a study.

All LULC projections used for this assessment are available at http://landcover-modeling.cr.usgs.gov. The computed predictor variables (covariates) used in this assessment, all range maps for 2001 and 2075 for each of the fifty modeled species, and a spreadsheet of all quantitative data reported in this paper are accessible at http://landcover-modeling.cr.usgs.gov/sdm.php. While this paper has focused on generalized results across the 50 modeled species, detailed model results for each of the 50 modeled species also are included herin. eBird data used as presence locations for this work may be obtained by through http://ebird.org.
